# Mutations of the *Genomes Uncoupled 4* Gene Cause ROS Accumulation and Repress Expression of Peroxidase Genes in Rice

**DOI:** 10.3389/fpls.2021.682453

**Published:** 2021-06-11

**Authors:** Rui-Qing Li, Meng Jiang, Jian-Zhong Huang, Ian Max Møller, Qing-Yao Shu

**Affiliations:** ^1^Hainan Institute of Zhejiang University, Yazhou Bay Science and Technology City, Sanya, China; ^2^College of Agronomy, Anhui Agricultural University, Hefei, China; ^3^National Key Laboratory of Rice Biology, Institute of Crop Sciences, Zhejiang University, Hangzhou, China; ^4^Department of Molecular Biology and Genetics, Aarhus University, Slagelse, Denmark

**Keywords:** GUN4, reactive oxygen species, anti-oxidative response, peroxidase, rice

## Abstract

The *Genomes Uncoupled 4* (*GUN4*) is one of the retrograde signaling genes in *Arabidopsis* and its orthologs have been identified in oxygenic phototrophic organisms from cyanobacterium to higher plants. GUN4 is involved in tetrapyrrole biosynthesis and its mutation often causes chlorophyll-deficient phenotypes with increased levels of reactive oxygen species (ROS), hence it has been speculated that GUN4 may also play a role in photoprotection. However, the biological mechanism leading to the increased ROS accumulation in *gun4* mutants remains largely unknown. In our previous studies, we generated an epi-mutant allele of *OsGUN4* (*gun4*^*epi*^), which downregulated its expression to ∼0.5% that of its wild-type (WT), and a complete knockout allele *gun4-1* due to abolishment of its translation start site. In the present study, three types of F_2_ plant derived from a *gun4-1/gun4^*epi*^* cross, i.e., *gun4-1/gun4-1*, *gun4-1/gun4^*epi*^* and *gun4^*epi*^/gun4^*epi*^* were developed and used for further investigation by growing them under photoperiodic condition (16 h/8 h light/dark) with low light (LL, 100 μmol photons m^–2^ s^–1^) or high light (HL, 1000 μmol photons m^–2^ s^–1^). The expression of *OsGUN4* was light responsive and had two peaks in the daytime. *gun4-1/gun4-1*-F_2_ seeds showed defective germination and died within 7 days. Significantly higher levels of ROS accumulated in all types of *OsGUN4* mutants than in WT plants under both the LL and HL conditions. A comparative RNA-seq analysis of WT variety LTB and its *gun4*^*epi*^ mutant HYB led to the identification of eight peroxidase (PRX)-encoding genes that were significantly downregulated in HYB. The transcription of these eight *PRX* genes was restored in transgenic HYB protoplasts overexpressing *OsGUN4*, while their expression was repressed in LTB protoplasts transformed with an *OsGUN4* silencing vector. We conclude that OsGUN4 is indispensable for rice, its expression is light- and oxidative-stress responsive, and it plays a role in ROS accumulation via its involvement in the transcriptional regulation of *PRX* genes.

## Introduction

The *Genomes Uncoupled 4* (*GUN4*) gene is one of six *GUN* genes that are involved in retrograde signaling pathways (Larkin et al., 2003). The *gun* mutants were originally identified in *Arabidopsis thaliana* as signal transduction mutants that uncouple their nuclear gene expression from chloroplast development (Susek et al., 1993). GUN4 is highly conserved in oxygenic photosynthetic plant species (Strand et al., 2003; Formighieri et al., 2012; Brzezowski et al., 2014; Li et al., 2017). GUN4 binds the product and substrate of Mg-chelatase, an enzyme that produces Mg-Proto, and activates Mg-chelatase, hence plays a regulatory role in chlorophyll biosynthesis in *Arabidopsis thaliana* (Larkin et al., 2003; Peter and Grimm, 2009; Adhikari et al., 2011; Richter et al., 2016), *Chlamydomonas reinhardtii* (Brzezowski et al., 2014), *Synechocystis* sp. (Sobotka et al., 2008; Chen et al., 2015) and rice (Zhou et al., 2012; Li et al., 2014a; Jiang et al., 2019).

*Genomes Uncoupled 4* is not an essential Mg-chelatase subunit *in vitro* or *in vivo* and hence is not essential for chlorophyll biosynthesis (Larkin et al., 2003; Davison et al., 2005; Verdecia et al., 2005; Peter and Grimm, 2009), but all *gun4* mutants have reduced chlorophyll contents throughout their life cycle. Indeed, mutations of *GUN4* and its orthologs not only resulted in reduced chlorophyll biosynthesis, but also greatly altered the content of tetrapyrrole intermediates. In the rice mutant HYB with *OsGUN4* being significantly downregulated, the content of heme and protoporphyrin IX (PPIX) was significantly increased while that of other tetrapyrrole metabolites such as Mg-protoporphyrin IX (Mg-PPIX) was significantly decreased (Li et al., 2017). Similar increases in PPIX content were reported for *gun4* mutants of *Synechocystis* sp. (Sobotka et al., 2008) and *C. reinhardtii* (Formighieri et al., 2012), while little or no increase was reported for *Arabidopsis* (Mochizuki et al., 2008).

An additional phenotype of *gun4* mutants is their increased photosensitivity, which has since led to the suggestion that GUN4 plays a role in photoprotection (Larkin et al., 2003; Adhikari et al., 2011; Formighieri et al., 2012). Larkin et al. (2003) proposed that GUN4 – the H subunit of Mg-Chelatase (ChlH) complexes could envelop and thereby shield PPIX and Mg-PPIX from collisions with O_2_ that might yield reactive oxygen species (ROS), and hence contribute to photoprotection. In contrast, Adhikari et al. (2011) developed *gun4* mutants with altered binding ability to porphyrin and investigated their ROS content, and demonstrated that the porphyrin-binding activity of GUN4 did not help attenuate ROS production. In *C. reinhardtii*, Brzezowski et al. (2014) proposed that GUN4 functions in photoprotection by preventing PPIX and Mg-PPIX from interacting with oxygen and thereby decreasing ROS production based on their study on the *gun4* and *chlD–1* mutants. However, Tabrizi et al. (2016) demonstrated that purified GUN4 together with oxidatively damaged ChlH increased the rate of PPIX-generated singlet oxygen (^1^O_2_) production in the light by a factor of 5 and 10 and therefore concluded that protoporphyrin bound to GUN4 was the actual ^1^O_2_ singlet oxygen generator. Also, in *C. reinhardtii gun4* mutants, Formighieri et al. (2012) observed perturbations in electron transport with a strongly decreased PSI-to-PSII ratio, which was accompanied by an enhanced activity of the plastid terminal oxidase (PTOX). They therefore proposed that GUN4 plays a physiological role in decreasing photosystem II excitation pressure. Thus, that the functioning mechanism of GUN4 in photoprotection and ROS homeostasis is still uncertain.

In plant chloroplasts, PSI and PSII reactive centers are the major sources of ROS (Asada, 1999; Møller et al., 2007), which in turn can damage the photosystems and disrupt electron transfer chain (ETC) (Laloi et al., 2004). One of the efficient anti-oxidant systems in the chloroplast involves enzymatic systems to remove ROS once formed (Dietz, 2016). ROS and redox regulation play important roles in the regulation of photosynthesis (Dietz and Hell, 2015). Two types of peroxidases (PRXs), the thiol-based and ascorbate-dependent peroxidases, are employed to link detoxification of peroxides and serve roles in redox regulation and retrograde signaling within and from the chloroplast (Dietz, 2016).

Obviously, the increased ROS accumulation observed in *gun4* mutants could also result from either increased production or decreased removal, or both. However, most studies on *gun4* mutants have focused on the binding ability of GUN4 to porphyrins and its role in ROS production. The possibility that GUN4 also plays a role in the regulation of ROS response genes such as PPX genes has never been addressed.

We identified an antioxidant response element (ARE) in the promoter of *OsGUN4* and demonstrated that its methylation dramatically downregulated its expression in the *xantha* mutant HYB, which produced a chlorophyll-deficient phenotype (Li et al., 2014a). We further demonstrated that the ^1^O_2_ level was significantly reduced in HYB compared with that of its wild-type (WT) parent Longtepu B (LTB) (Li et al., 2017). These observations led us to propose that GUN4 plays a role in the regulation of ROS response genes, which may explain the increased ROS accumulation in *gun4* mutants. In the present study, we first examined the response of *OsGUN4* expression to light intensity and oxidative stress and assessed ROS accumulation in *osgun4* mutants; we then performed RNA-seq and identified differentially expressed PRX genes and finally; we tested the effect of OsGUN4 on the expression of PRX genes in protoplasts by silencing and complementing *OsGUN4*. We show that *OsGUN4* expression is light- and oxidative-stress responsive, and OsGUN4 plays a role in ROS accumulation via its involvement in the transcriptional regulation of *PRX* genes in rice.

## Materials and Methods

### Plant Materials

From wild-type *indica* variety Longtepu B (LTB), we previously developed a *xantha* mutant line, Huangyu B (HYB) via gamma ray mutagenesis (Zhou et al., 2006). HYB carries an epi-mutant allele of *OsGUN4* (hereafter *gun4*^*epi*^), which downregulates the expression of *OsGUN4* to ∼ 0.5% that of LTB (Li et al., 2014a). Further, through gamma ray irradiation, our previous study also succeeded in generating and identifying a few *OsGUN4* mutant alleles in the background of a *japonica* line GS113 by crossing HYB to GS113 M_2_ plants (Li et al., 2014a). Among them, the *gun4-1* allele is a complete knockout mutation because its translation initiation codon (ATG) is completely deleted (and thus no OsGUN4 protein is expected to be translated) (Li et al., 2014a). In the present study, the F_2_ seeds of the F_1_ hybrid plant of GS113 (*gun4-1*)/HYB (*gun4*^*epi*^) were used for various investigations, together with LTB and HYB (Supplementary Figure 1).

For easy management, germinated seeds were first grown in plastic trays with soils taken from paddy fields (surface, not sterilized) of Zhejiang University Experimental Farm at 30°C for 7 days in growth rooms with LED (light emitting diode) under 16 h/8 h light/dark, at low light (LL, 100 μmol photons m^–2^ s^–1^) or high light (HL, 1000 μmol photons m^–2^ s^–1^) conditions. For assessing the effect of light intensity on seedling growth and gene expression, seven-day old seedlings grown on soil were transferred to 1× MS liquid medium (Murashige and Skoog, 1962) and grown under either LL or HL conditions for another 7–28 days. For assessment of H_2_O_2_ effect, seedlings that had been grown on soil (7 days) and 1× MS medium (21 days) under LL were transferred to 1× MS liquid medium with or without 1 mM H_2_O_2_ and grown for another 7 days under LL. Liquid medium was replaced every 3 days.

For genotyping of the F_2_ seedlings, genomic DNA was extracted from leaf segments and subject to high resolution melting (HRM) curve analysis with primers of 5′-TTACCGG CAGGCCGACGAGA-3′ and 5′-TGCCCAGGAGCTGTGTCC CT-3′ according to Tan et al. (2016).

For investigation of diurnal expression of *OsGUN4*, seedlings of LTB and Nipponbare (*japonica*) were grown in soil under HL conditions (16 h/8 h light/dark) for 35 days after germination (DAG), and their leaves were sampled every 2 h for RNA extraction within a 24-h period.

### Quantification of Chlorophylls and Tetrapyrrole Metabolites

The concentrations of chlorophylls were determined using 0.3 g fresh leaf tissues following a published method (Peter and Grimm, 2009). For measurement of PPIX, Mg-PPIX and protochlorophyllide (pchlide), 0.3 g fine powder of liquid nitrogen-ground leaves were immediately mixed with pre-cold alkaline acetone containing 0.1 N NH_4_OH (9:1; v/v), and centrifugated at 16,000 *g* for 5 min. Subsequently, the supernatants were collected for extraction of PPIX (Papenbrock and Grimm, 2001), Mg-PPIX (Papenbrock and Grimm, 2001), and pchlide (Koski and Smith, 1948). The contents of PPIX, Mg-PPIX and pchlide were determined with commercial enzyme-linked immunosorbent assay (ELISA; Engvall and Perlmann, 1971) kits (Jiangsu Jingmei Biotechnology Co., Ltd., Yancheng, China) following the manufacturer’s instructions.

### Measurement of Singlet Oxygen, Hydrogen Peroxide and Total ROS

The concentration of singlet oxygen (^1^O_2_) was determined using the SOSG (singlet oxygen sensor green) method (Hideg et al., 2002). Briefly, the liquid nitrogen homogenized power of 0.3 g leaf samples were suspended with pre-cold lysis buffer (GENMED Scientifics Inc., Shanghai, China), and then immediately centrifuged at 4°C for 10 min (5000 *g*). Subsequently, SOSG (GENMED Scientifics Inc.) was added to the supernatant to a final concentration of 10 μM. The fluorescence spectra were detected at excitation of 485 nm and emission of 520 nm using a fluorescence spectrophotometer (359S, Lengguang Tech., Shanghai, China).

The hydrogen peroxide (H_2_O_2_) content was measured according to the method of Rokebul Anower et al. (2017) with minor modifications. 0.3 g leaf tissues were ground in liquid nitrogen and then immediately mixed with pre-chilled Krebs–Ringer phosphate (145 mM NaCl, 5.7 mM sodium phosphate, 4.86 mM KCl, 0.54 mM CaCl_2_, 1.22 mM MgSO_4_, 5.5 mM glucose, pH 7.35). Subsequently, the homogenate was centrifuged at 12,000 *g* for 20 min at 4°C, and then the supernatants were incubated with 50 μM Amplex^®^ Red reagent (10-acetyl-3,7-dihydrophenoxazine, Thermo Fisher Scientific, Shanghai, China) and 0.1 U/mL horseradish peroxidase (HRP, Thermo Fisher Scientific, Shanghai China) at 37°C for 10 min away from light. The fluorescence was measured at 530 nm under irradiation of 590 nm light for excitation by using a VersaFluor Fluorometer (Bio-Rad, Hercules, CA, United States) and calculated based on a standard curve constructed using known concentrations of H_2_O_2_.

The total ROS concentration was determined according to the method of Joo et al. (2005) with modifications. In brief, for assessment of ROS in seedlings, the above-ground part of seedlings was weighed, quickly frozen and ground into powder in liquid nitrogen, and immediately suspended in 10 mM Tris-HCl buffer (pH 7.3) for extraction. The mixture was centrifuged at 16,000 *g* for 5 min, and after collecting the supernatant, the pellet was suspended with buffer and centrifuged for another 5 min. For assessment of ROS in protoplasts, the transfected protoplasts containing 4 × 10^4^ protoplasts/μL were added with 10 mM H_2_DCFDA (Thermo Fisher Scientific, Waltham, MA, United States) at 4°C in DMSO.

The supernatant was used for ROS measurement by adding 10 mM H_2_DCFDA (Thermo Fisher Scientific, Waltham, MA, United States) at 4°C in DMSO. The fluorescence was measured at 530 nm under irradiation of 488 nm light for excitation by using a VersaFluor fluorometer (Bio-Rad, Hercules, CA, United States) with six biological replicates. The ROS content, expressed as relative fluorescence units, was obtained from six successive measurements and is given as units per milligram of fresh weight.

### Quantitative Real-Time PCR Analysis

Total RNA was extracted from fresh leaves or protoplasts using the Qiagen Spin Plant RNA Mini Kit (Qiagen, Hilden, Germany). cDNA was reverse transcribed from 1 *μ*g total RNA with oligo-dT_18_ Primer using GoScript^TM^ Reverse Transcription System Kit (Promega, WI, United States) according to the manufacturer’s instructions. Quantitative real-time PCR (qRT-PCR) was performed using a SYBR Green GoTaq^®^ qPCR Master Mix containing ROX as internal control (Promega, WI, United States). All qRT-PCRs used the following cycling conditions: 10 min at 95°C, 40 cycles of 30 s at 94°C, 30 s at 55°C and 60 s at 72°C. Relative mRNA abundance was analyzed with the rice *Ubiquitin g*ene as internal reference using the 2^–ΔΔCt^ method (Livak and Schmittgen, 2001). Primers used for qRT-PCR are listed in Supplementary Table 1.

### RNA Sequencing

Total RNA of LTB and HYB was extracted from fresh leaf tissues of 35 DAG seedlings using the Qiagen Spin Plant RNA Mini Kit (Qiagen, Hilden, Germany). cDNA libraries were constructed with oligo-dT_18_ Primer using GoScript^TM^ Reverse Transcription System Kit (Promega, WI, United States) according to the manufacturer’s instructions, and were subsequently sequenced on an Illumina Hiseq 2000 platform (Beijing Novogene Bioinformatics Technology Co., Ltd., Beijing, China). For mapping, the raw reads were cleaned by removing adapter sequences using the Cutadapt (v1.11) software and then aligned to the reference genome sequences (IRGSP-1.0^1^, 09/17/2016) by using the Tophat v2.0.9 program with *E*-value < 10^–5^ as cut-off point (Trapnell et al., 2012). For detection of differentially expressed genes (DEGs), the DESeq package (ver 2.1.0) was used with a false discovery rate (FDR) ≤ 0.05 and the absolute value of the log_2_ (fold change ≥ 2) with RPKM ≥ 1 as the threshold to determine significant differences of gene expression. Gene ontology (GO) enrichment was conducted according to PANTHER Overrepresentation Test (released 20210224) by using GO Ontology database (released 02/01/2021) using *Oryza sativa* database as a reference list, and was visualized with ggplot2 R package. KEGG pathways were analyzed with a FDR ≤ 0.05 as significant levels of differential expression. DEGs potentially related to ROS homeostasis were identified and subjected to RT-q PCR analysis using gene-specific primers (Supplementary Table 1).

### Functional Complementation/Overexpression and Silencing of Genes in a Protoplast System

For construction of *OsGUN4* complementary expression vector, a DNA fragment encompassing the full open reading frame of *OsGUN4* and its upstream (2447 bp) and downstream (518 bp) sequences were amplified from genomic DNA of Nipponbare using primers OGUN4F and OGUN4R (Supplementary Table 1), which was cloned into a pUCm-T vector (Sangon Biotech, Shanghai, China). The recombinant pUCm-T vector was then digested with *Nco*I/*Hind*III and the target fragment was cloned into binary vector pCAMBIA 1301 using the same restriction enzymes. The ultimate vector is named pOsGUN4-C.

For construction of *OsPRX39* overexpression vector, the full open reading frame of *OsPRX39* (999 bp) sequences were amplified from genomic DNA of Nipponbare with the primers OPRX39F/OPRX39R (Supplementary Table 1), which was cloned into a pUCm-T vector (Sangon Biotech, Shanghai, China), and the recombinant vector was digested with *NcoI/EcoRI*. Subsequently, the target fragment was simultaneously cloned into the digested binary vector of pCAMBIA 1301 (by using *NcoI/EcoRI*), the *OsPRX39* overexpression vector was thus constructed and named p1301-Ubi-PRX39.

For silencing of *OsGUN4*, the hairpin RNA (hpRNA) down-regulating system was used and a silencing vector was constructed according to Li et al. (2014b). An *OsGUN4* fragment (+562 to +761 bp downstream of the ^+1^ATG), containing part of the coding sequence and a partial 3′ UTR was amplified using primers RGUN4F and RGUN4R (Supplementary Table 1) and cloned into a pUCm-T vector. Two fragments containing the *OsGUN4* fragment were cloned from the recombinant pUCm-T vector by double restriction with *NotI*/*BamHI* and *XhoI*/*KpnI*, respectively. They were then sequentially ligated into pBSSK-IN in the sense and antisense orientations. The *OsGUN4* hairpin structure in the recombinant pBSSK-IN was introduced into pCAMBIA1301-35S using restriction enzymes *KpnI* and *SacI*. The recombinant pCAMBIA1301-35S vector is named pOsGUN4-I.

Using the slightly modified enzymolysis method (Zhang et al., 2011), protoplasts of LTB and HYB were extracted from 35 DAG seedlings grown under LL conditions and used for transient expression analysis. They were transformed with the *OsGUN4* complementation/overexpression or silencing vectors with PEG-mediated transformation according to Bart et al. (2006). Briefly, 100 μL solution containing 4 × 10^4^ protoplasts/μL was transformed with 10 μg plasmids and incubated in W5 solution (2.4 × 10^4^ protoplasts/μL) in darkness for about 1 h. The transformed protoplasts were then cultured at low light or high light for another 6 h before RNA extraction.

### Statistical Analysis

All experiments were performed with six biological replicates. Values are expressed as means ± standard deviations. Comparisons of data from different groups were analyzed using ANOVA test followed by the Tukey’s Multiple Comparison Test with *P* < 0.05.

## Results

### *OsGUN4* Expression Is Responsive to Light Intensity and Oxidative Stress

**FIGURE 1:**
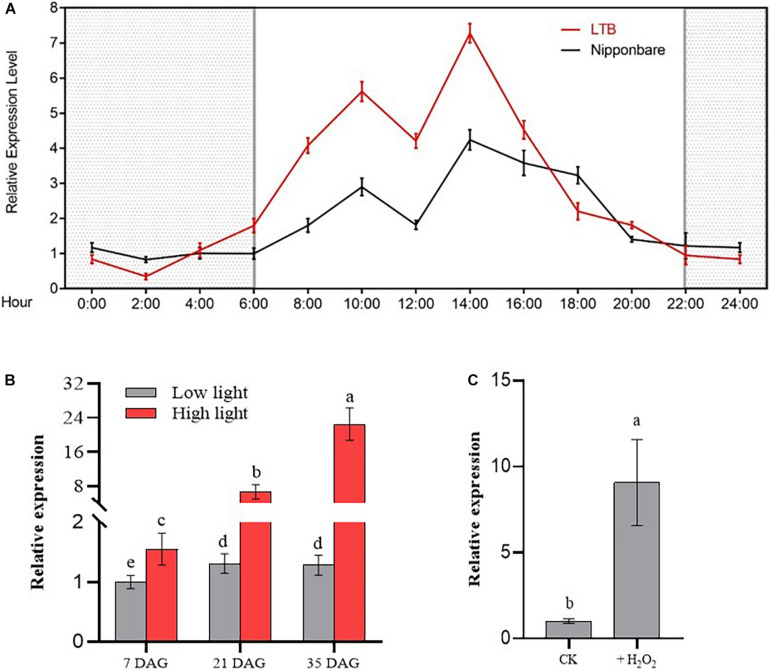
Relative expression of *OsGUN4* in seedlings 35 days after germination. **(A)** Variation of *OsGUN4* expression in wild-type *indica* rice Longtepu B (LTB) and *japonica* rice Nipponbare across a 24-h period grown in growth room in photoperiodic conditions (16 h light/8 h dark). The light intensity was 1000 μmol m^–2^ s^–1^ and the temperature was set at a constant 30°C. Samples were taken every 2 h from 6:00 am when light was turned on. Relative expression is reported relative to that of Nipponbare at 6:00 am, which was assigned a value of 1. **(B)** Relative expression of *OsGUN4* in LTB seedlings at 35 days after germination (DAG) grown under either high light (1000 μmol m^–2^ s^–1^) or low light (100 μmol m^–2^ s^–1^) with temperature set at a constant 30°C. Relative expression is reported in relative to that of LTB seedlings at 7 DAG, which was assigned a value of 1. **(C)** Relative expression of *OsGUN4* in LTB seedlings subjected to oxidative stress. Seedlings of 7 DAG were first grown in 1× MS liquid medium for 21 days and then further grown with or without 1 mM H_2_O_2_ for another 7 days. All seedlings were under low light conditions. Relative expression is reported in relative to that of that control, which was assigned a value of 1.

To detect the daily variation of *OsGUN4* expression, we first we investigated its relative expression in seedlings of *indica* variety LTB and *japonica* variety Nipponbare at different times during a 24-h period (Figure 1A). We observed that the expression of *OsGUN4* was lower at night and quickly increased after day-break at 06 00 h. Two peaks of expression were observed at 10 00 and 14 00 h for both varieties. The expression gradually decreased in the afternoon and remained at a low level at night. These results suggest that the expression of *OsGUN4* had the nature of diurnal oscillation, and the relatively reduced expression at the mid-day suggests that its expression is also subject to internal physiological changes.

To examine the responsiveness of *OsGUN4* to light intensity, the abundance of *OsGUN4* mRNA transcripts was examined for seedlings grown under LL and HL conditions at 7, 21, and 35 days after germination (DAG) (Figure 1B). When grown under LL conditions, the expression of *OsGUN4* was low and hardly increased with plant age (Figure 1B and Supplementary Table 2). Under HL conditions, the *OsGUN4* expression level significantly increased with seedling age, and was much higher than that of seedlings grown under LL conditions at all ages (Figure 1B). For example, at 7 DAG, the *OsGUN4* expression level in seedlings under HL conditions was 1.53 that of seedlings under LL, while at 35 DAG, the relative value increased to 17.49. Thus, *OsGUN4* expression was responsive to light intensity and increased with seedling age.

To examine its responsiveness to oxidative stress, the expression of *OsGUN4* was examined in seedlings grown for 7 days in medium with 1 mM H_2_O_2_. The *OsGUN4* expression level was significantly higher (8.8-fold) in seedlings treated with H_2_O_2_ than in the control plants (Figure 1C and Supplementary Table 2), indicating a response to oxidative stress.

### Impaired Growth of the *OsGUN4* Mutant Seedlings

**FIGURE 2:**
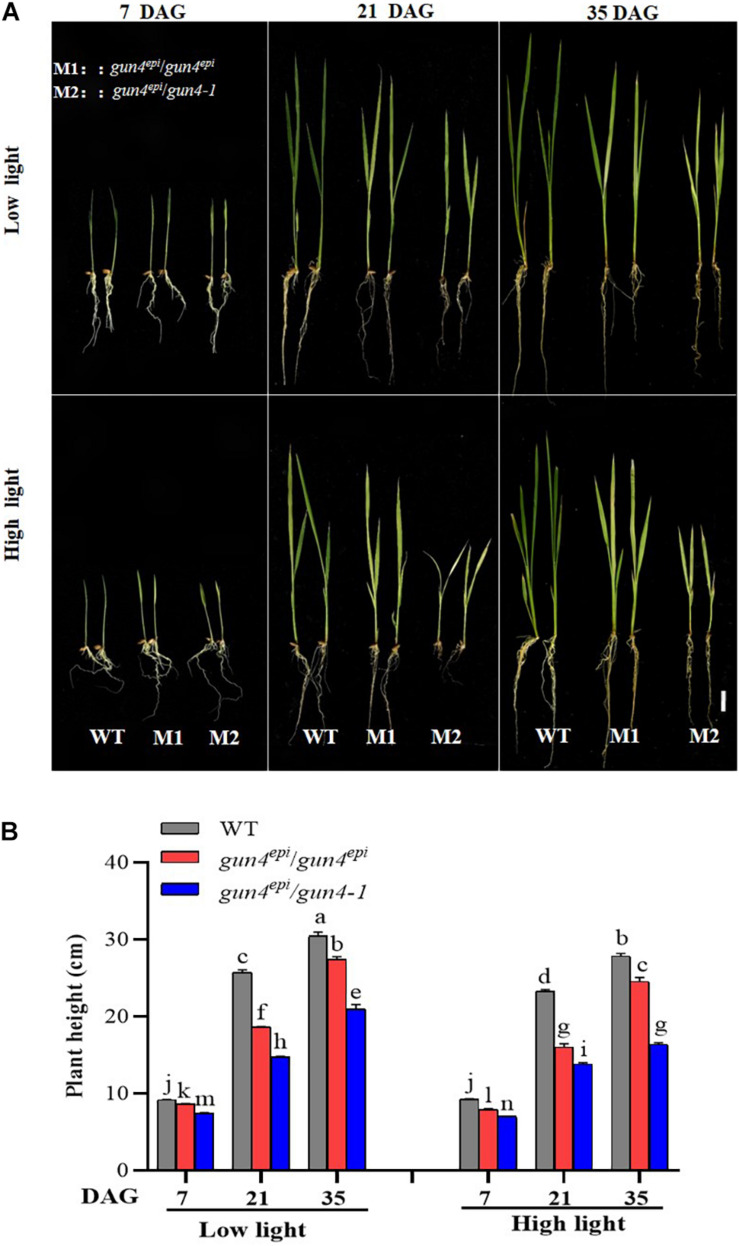
Effects of high light on growth of the *OsGUN4* mutants. **(A)** The phenotype and **(B)** plant height of wild-type (WT; c.v. LTB) and F_2_ seedlings derived from *gun4*^*epi*^/*gun4-1* F_1_ plants at 7, 21, and 35 days after germination (DAG). Values are means ± SD (*n* = 6) and analyzed for significant differences by two-way ANOVA followed by the Tukey’s multiple comparison test, *P* < 0.05. Scale bar = 3 cm. For the background information of the F_1_ plants, see Supplementary Figure 1.

Down-regulation of *OsGUN4* is known to result in chlorophyll deficiency and reduced plant growth, but it was not known whether rice could survive a complete *OsGUN4* knockout under different light conditions. During the germination stage, we observed that a high percentage of F_2_ seeds derived from the *gun4-1/gun4^*epi*^* F_1_ plants could not develop into complete seedlings and died within 7 DAG (Figure 2 and Supplementary Figure 2A).

To distinguish the effect of the *gun4*^*epi*^ and *gun4-1* mutations on seed germination and seedling growth, we performed an HRM-based genotyping (Supplementary Figure 1B). It was revealed that all the early dying germinated seeds belonged to the *gun4-1/gun4-1* genotype, suggesting OsGUN4 is indispensable for rice seedling growth. The *gun4^*epi*^/gun4^*epi*^* and *gun4^*epi*^/gun4-1* F_2_ seedlings were further grown under different conditions and their growth and ROS content were measured.

Most F_2_ seedlings with the *gun4^*epi*^/gun4-1* genotype survived, but their growth was significantly slower and weaker than the WT and *gun4^*epi*^/gun4^*epi*^* ones, particularly under the HL conditions (Figure 2A; Supplementary Figure 2B; and Supplementary Table 3). Under HL conditions, the *gun4^*epi*^/gun4-1* F_2_ seedlings were significantly shorter than the *gun4^*epi*^/gun4^*epi*^* ones (Figure 2B), suggesting that there is a dosage effect of the *gun4*^*epi*^ allele on seedling growth.

### Changes in Tetrapyrrole Metabolite Levels

**FIGURE 3:**
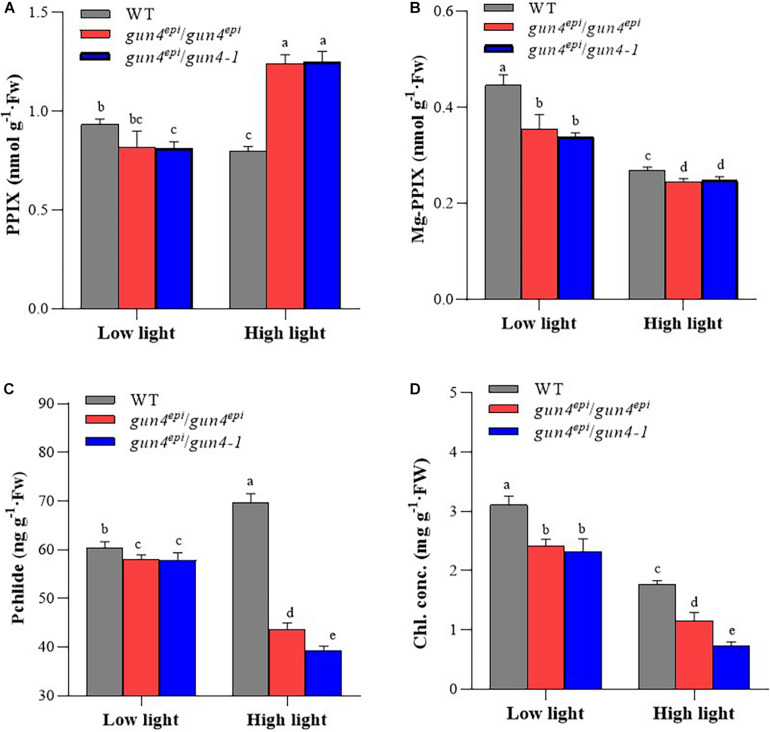
The concentration of tetrapyrrole metabolites in wild-type (WT) and *OsGUN4* mutant rice seedlings. Seedlings were at 35 days after germination (DAG) grown under low (100 μmol m^–2^ s^–1^ photons) or high (1000 μmol m^–2^ s^–1^ photons) light conditions. WT, wild-type variety Longtepu B, *gun4*^*epi*^/*gun4*^*epi*^ and *gun4*^*epi*^/*gun4-1* are F_2_ seedlings derived from the *gun4*^*epi*^/*gun4-1* F_1_ plants. PPIX, protoporphyrin IX; Mg-PPIX, Mg-protoporphyrin IX; Pchlide, protochlorophyllide; Chl, chlorophyll. Values are means ± SD (*n* = 6) and analyzed for significant differences by two-way ANOVA followed by the Tukey’s multiple comparison test, *P* < 0.05. For the background information of the F_1_ plants, see Supplementary Figure 1.

To assess the effect of *OsGUN4* mutations on tetrapyrrole metabolism, the concentrations of chlorophyll (Chl) and three key tetrapyrrole metabolites was measured in 35 DAG seedlings grown under LL and HL conditions (Figure 3 and Supplementary Table 4). Under LL conditions, in consistent with phenotypical changes, although the concentration of Chl and tetrapyrrole metabolites was significant lower in the *OsGUN4* mutants than the WT, the differences were very limited between mutants and the WT, with the maximum difference being observed for Chl (<30%, Figure 3D); No significantly differences were detected for any of the metabolites between the F_2_ seedlings of *gun4^*epi*^/gun4^*epi*^* and *gun4^*epi*^/gun4-1* (Figure 3).

Under HL condition, the metabolite concentration differences became much more pronounced between the two mutants and the WT (Figure 3). PPIX is the tetrapyrrole metabolite that can be diverted into both heme (through Fe-branch) and Chl (through Mg-branch), its concentration was significantly higher (+60%) in the two mutants than in the WT (Figure 3A). However, the metabolites in the Mg-branch were significantly reduced in both mutants (Figures 3B–D). The two mutants had 89% of the Mg-PPIX concentration in the WT (Figure 3B). The reduction of pchlide and Chl contents was more dramatic; the F_2_ seedlings of *gun4^*epi*^/gun4^*epi*^* and *gun4^*epi*^/gun4-1* had pchlide content only 63 and 56% that of the WT, and Chl content only 65 and 41% that of the WT, respectively (Figures 3C,D).

When WT seedlings were moved from LL to HL conditions, the concentration of PPIX, Mg-PPIX and Chl was significantly reduced, while that of pchlide significantly increased. In the two *OsGUN4* mutants, the concentrations of all metabolites except PPIX significantly decreased (Figure 3). Thus, *OsGUN4* mutations also changed the responsiveness of tetrapyrrole metabolism to light intensity.

### Elevated Accumulation of ROS in *OsGUN4* Mutant Seedlings

**FIGURE 4:**
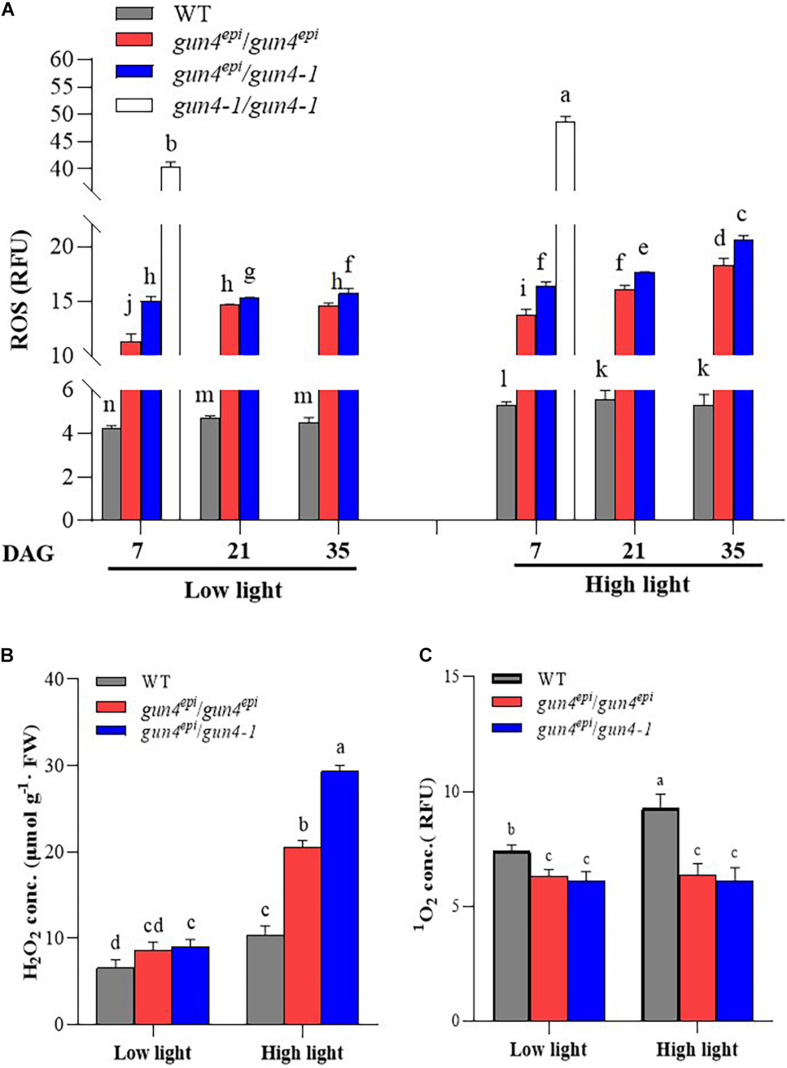
Effects of high light on the accumulation of reactive oxygen species (ROS) in the *OsGUN4* mutants. **(A)** The total reactive oxygen species (ROS) at 7, 21, and 35 days after germination (DAG), as well as concentrations of **(B)** hydrogen peroxide (H_2_O_2_) and **(C)** singlet oxygen (^1^O_2_) in rice seedlings at 35 DAG. WT, wild-type variety Longtepu B, *gun4*^*epi*^/*gun4*^*epi*^ and *gun4*^*epi*^/*gun4-1* are F_2_ seedlings derived from the *gun4*^*epi*^/*gun4-1* F_1_ plants. The data were analyzed with two-way ANOVA followed by the Tukey’s multiple comparison test, *P* < 0.05. For the background information of the F_1_ plants, see Supplementary Figure 1.

To further examine whether the repressive effect of *OsGUN4* mutations on seedling growth and tetrapyrrole metabolism was ROS-dependent, we measured the concentrations of total ROS, H_2_O_2_ and ^1^O_2_ in the F_2_ and WT seedlings at different growth stages under LL and HL conditions. Significantly greater total ROS and H_2_O_2_ contents, but lower ^1^O_2_ content, was observed in the mutant F_2_ seedlings than the WT line, irrespective of the growth conditions and mutant genotype (Figure 4 and Supplementary Table 5).

For 7 DAG seedlings, we were able to measure the total ROS level in all three types of mutant seedlings (Figure 4A) and observed that the total ROS level in the *gun4-1*/*gun4-1* seedlings, irrespective of growth condition, was much higher than those in the WT and other two mutants. For example, under HL conditions, *gun4-1*/*gun4-1* F_2_ seedlings accumulated 9.18, 3.54, and 2.97 times as much total ROS than WT, *gun4^*epi*^/gun4^*epi*^* and *gun4*^*epi*^/*gun4-1*, respectively (Figure 4A). Because *gun4-1*/*gun4-1* F_2_ seedlings did not survive beyond 7 DAG, ROS content could only be measured on seedlings of the other two mutants at 21 and 35 DAG. The *gun4*^*epi*^/*gun4-1* F_2_ seedlings accumulated significantly higher levels of total ROS than the *gun4^*epi*^/gun4^*epi*^* ones. Comparatively, both types of mutant seedlings contained far more ROS than the WT at all time point, i.e., the *gun4^*epi*^/gun4^*epi*^* F_2_ seedlings accumulated 2.7–3.3 times, under the LL conditions, and 2.6–3.5 times under the HL conditions (Figure 4A) more ROS than the WT (Figure 4A), respectively.

The total ROS level in the *gun4*^*epi*^/*gun4-1* F_2_ seedlings increased with seedling age under both the LL and HL conditions. A similar trend was observed in *gun4^*epi*^/gun4^*epi*^* F_2_ seedlings grown under the HL conditions (Figure 4A). At a given growth stage, significantly more ROS accumulated in seedlings grown under HL conditions than under LL conditions irrespective of genotype (Figure 4A).

To differentiate different types of ROS, the seedlings at 35 DAG were further assessed for the level of H_2_O_2_ and ^1^O_2_. Significantly higher levels of H_2_O_2_ were observed in mutant seedlings than that in the WT; the differences became more significant under HL conditions. The levels of H_2_O_2_ in *gun4^*epi*^/gun4^*epi*^* and *gun4^*epi*^/gun4-1* F_2_ seedlings were 2.5- and 3.2-fold those of the WT, respectively (Figure 4B and Supplementary Table 6). Both *gun4^*epi*^/gun4^*epi*^* and *gun4^*epi*^/gun4-1* F_2_ seedlings accumulated significantly lower levels of ^1^O_2_ (66–87%) compared to the WT, irrespective of growth conditions (Figure 4C and Supplementary Table 6). Furthermore, HL treatment induced an increase in ^1^O_2_ content by 25% in WT, while no significant change was detected in the mutants under any condition (Figure 4C).

### Identification of Differentially Expressed Genes Encoding ROS-Degrading Enzymes by Transcriptome Sequencing

**FIGURE 5:**
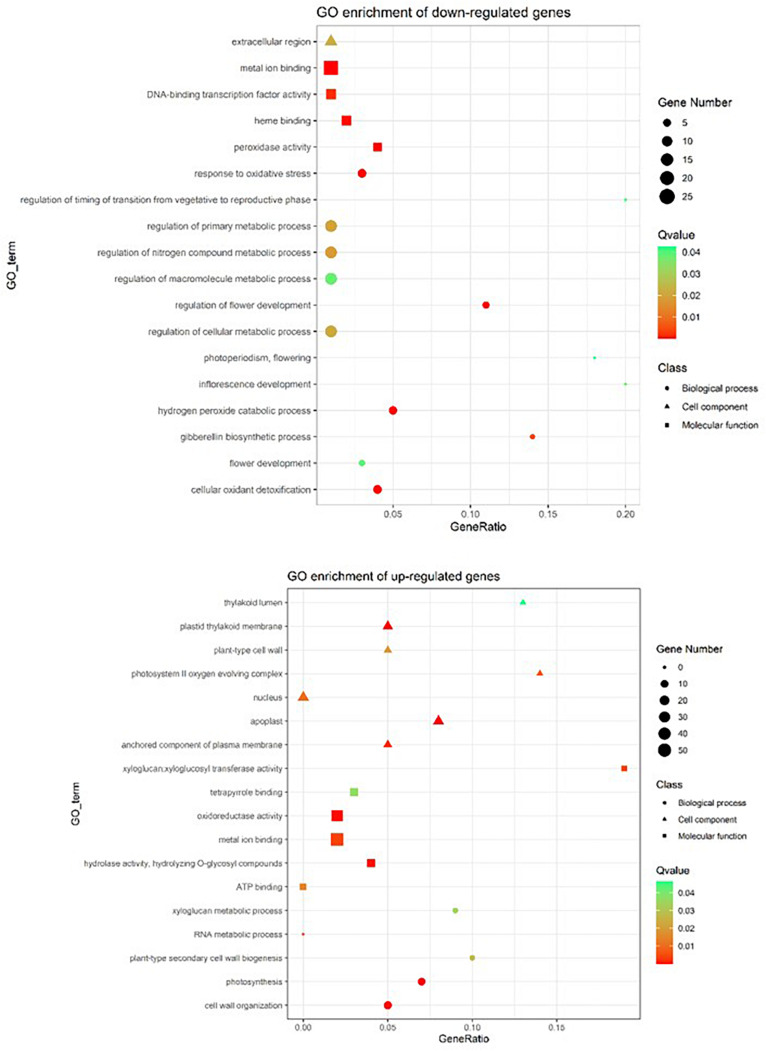
GO enrichment of differentially expressed genes in the epigenetic *gun4* mutant HYB as compared with its wild-type parent LTB, revealed by RNA-seq in 35 DAG leaves. Gene ontology (GO) enrichment was conducted according to PANTHER Overrepresentation Test (released 20210224) by using GO Ontology database (released 02/01/2021) using Oryza sativa database as a reference list. False Discovery Rate was applied for statistics. The ggplot2 was used to provide visualizations for enrichment results.

To examine the effect of the *gun4*^*epi*^ mutation on overall gene expression, the transcriptomes of LTB and its epigenetic mutant HYB at 35 DAG were sequenced (Supplementary Tables 7, 8). Besides, a total of 468 differentially expressed genes (DEGs) were identified between HYB and LTB, with 203 genes being up-regulated and 265 down-regulated in HYB. GO analysis showed genes involved in peroxidase activity, response to oxidative stress were down-regulated (Figure 5). Further examination revealed that eight peroxidase (PRX)-encoding genes were among the down-regulated genes.

### Transcription of 8 *PRX* Genes Is Responsive to Oxidative Stress in WT but Significantly Repressed in *OsGUN4* Mutants

**FIGURE 6:**
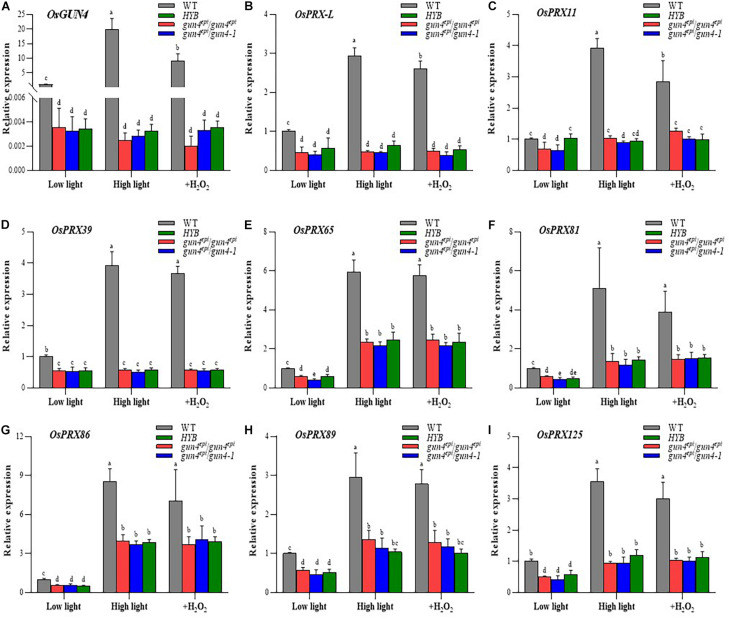
Relative expression levels of *OsGUN4* and 8 peroxidase-encoding genes. Relative expression levels of *OsGUN4* **(A)** and 8 peroxidase (PRX)-encoding genes **(B–I)** in 35-day-old seedlings. WT, wild-type variety LTB; HYB, the epigenetic *OsGUN4* mutant LTB (*gun4*^*epi*^/*gun4*^*epi*^) of LTB; *gun4*^*epi*^/*gun4*^*epi*^ and *gun4*^*epi*^/*gun4-1* are F_2_ seedlings derived from the *gun4*^*epi*^/*gun4-1* F_1_ plants. Seedlings were grown under low (100 μmol m^–2^ s^–1^ photons) or high (1000 μmol m^–2^ s^–1^ photons) light conditions, or in medium with 1 mM exogenous H_2_O_2_ (+H_2_O_2_) under low light condition. Expression levels are reported relative to that of the WT grown under low light, which was assigned a value of 1. Values are means ± SD (*n* = 6) and analyzed for significant differences by two-way ANOVA followed by the Tukey’s multiple comparison test, *P* < 0.05. For more detail of seedling growth, see M&M.

We already demonstrated that transcription of *OsGUN4* is responsive to high HL conditions and H_2_O_2_ supplement (Figures 1B,C), this type of response of *OsGUN4* expression is no longer present in HYB seedlings: *OsGUN4* expression is extremely low irrespective of treatment, and no significant differences were observed for *OsGUN4* transcript abundance in HYB and the two F_2_ mutant seedlings (Figure 6A). These results indicated that the *OsGUN4* mutations not only dramatically reduced its transcription but also abolished its response to oxidative stresses caused by HL and H_2_O_2_. Similar performance of the mutant F_2_ seedlings to HYB indicated that the mutation effects were independent of genetic background.

qRT-PCR analyses demonstrated that the 8 PRX genes downregulated in HYB were all responsive to HL and exogenous H_2_O_2_ treatment in the WT (Figure 6 and Supplementary Table 9). The shift from LL to HL resulted in significant increases of transcripts abundance, i.e., from 2.96-times (*OsPRX89*, Figure 6H) to 8.54-times (*OsPRX86*, Figure 6G); Exogenous H_2_O_2_ treatment also generated similar increases (but slightly less than HL shift).

The transcriptional response of the 8 PRX genes to HL and H_2_O_2_ treatment seemed to be either significantly reduced or abolished in HYB and the mutant F_2_ seedlings (Figures 6B–I). Three genes (*OsPRX-*L, *OsPRX* 11 and *OsPRX*39) were no longer responsive to HL and H_2_O_2_ treatment (Figures 6B–D), and the remaining genes became significantly less responsive (Figures 6E–I).

### Silencing and Complementation of *OsGUN4* Expression and Their Effect on Transcription of 8 PRX Genes in a Protoplast System

To test whether the expression differences of the 8 PRX genes in WT and mutant seedlings are truly due to *OsGUN4* mutation, LTB protoplasts were transformed with the *OsGUN4* interference vector pOsGUN4-I and HYB protoplasts were transformed with the *OsGUN4* complementary vector pOsGUN4-C. The transformed protoplasts were subjected to LL and HL treatment and transcription of *OsGUN4* and the 8 PRX genes were then analyzed by q RT-PCR.

**FIGURE 7:**
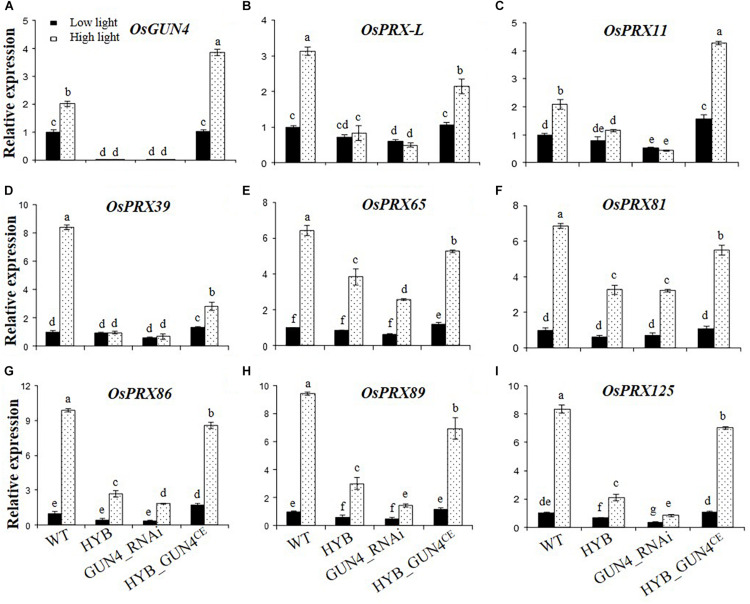
Relative expression levels of *OsGUN4* and 8 peroxidase-encoding genes in rice protoplasts. Relative expression levels of *OsGUN4* **(A)** and 8 peroxidase-encoding genes **(B–I)** in protoplasts of the wild-type (WT; c.v. LTB) and its *OsGUN4* epigenetic mutant HYB, and in LTB protoplasts transformed with an RNAi vector of *OsGUN4* (GUN4_RNAi) and HYB protoplasts with an *OsGUN4* complementary vector (HYB_GUN4^CE^). Protoplasts were prepared from leaves of seedlings 35 days after germination (DAG) grown under low light (100 μmol m^–2^ s^–1^) and transformed with interference or complementary vectors. Transformed protoplasts were initially kept under dark for 1 h and then grown under low (100 μmol m^–2^ s^–1^) or high light (1000 μmol m^–2^ s^–1^) for 6 h, before subject to total RNA extraction. Expression levels are reported relative to that of the WT protoplasts grown at low light, which was assigned a value of 1. Values are means ± SD (*n* = 6) and analyzed for significant differences by two-way ANOVA followed by the Tukey’s multiple comparison test, *P* < 0.05.

The expression pattern of *OsGUN4* in protoplasts seemed similar to that in seedlings, i.e., its expression was extremely low in HYB and did not increase after HL treatment, in sharp contrast to what was observed in WT (Figure 7A). Importantly, we succeeded in repressing *OsGUN4* expression by transforming LTB protoplasts with a silencing vector (GUN4_RNAi) and restoring its expression by transforming HYB protoplasts with a complementation vector (GUN4^CE^; Figure 7A).

The transcription of *OsGUN4* and the 8 PRX genes was significantly affected by light intensity and *OsGUN4* genotype (Supplementary Table 10). The native WT *OsGUN4* promoter was used for the construct of both vectors, hence *OsGUN4* expression in HYB protoplasts transformed with the complementation vector was also responsive to HL. In WT protoplasts transformed with OsGUN4-RNAi, transcription of *OsGUN4* was downregulated to the level similar to that in HYB at both LL and HL, while HYB protoplasts transformed with GUN4^CE^, the expression level of *OsGUN4* was restored to nearly the same level of WT at LL, and to higher levels at HL (Figure 7A).

In both WT and HYB_GUN4^CE^ protoplasts, transcription of all the 8 PRX genes was significantly increased after HL treatment. In HYB protoplasts transformed with GUN4^CE^, the transcription level of *OsPRX* genes was similar to or significantly higher than that of WT at LL, but did not restore to the same level as in the WT except *OsPRX11* at HL (Figures 7B–I). Thus, the transcriptional response of PRX genes to light fluence shift was restored to different degrees in *OsGUN*4-complemented HYB protoplasts.

In both HYB and GUN4_RNAi protoplasts, either no such increase (*OsPRX-L*, *OsPRX11* and *OsPRX39*) or limited increase of transcription was observed for the other genes, similar to the pattern observed in seedlings. In LTB protoplasts transformed with the interference vector, the expression level of *OsPRXs* was reduced to 10–44% that of untransformed protoplasts and was very similar to that observed in HYB, their response to HL was either abolished (*OsPRX-*L, *OsPRX* 11 and *OsPRX*39, Figures 7B–D) or significantly repressed (*OsPRX*65, *OsPRX* 81 and *OsPRX*86, *OsPRX* 89 and *OsPRX*125, Figures 7E–I).

These results demonstrate that the protoplast expression system worked well for interference and complementation of *OsGUN4* mutation, and that there is a cause-and-effect relationship between *OsGUN4* mutation and altered expression and response of the 8 *PRX* genes to a light fluence shift and H_2_O_2_ treatment.

### Effect of *OsGUN4* and *OsPRX39* Expression on ROS Accumulation in Protoplasts

**FIGURE 8:**
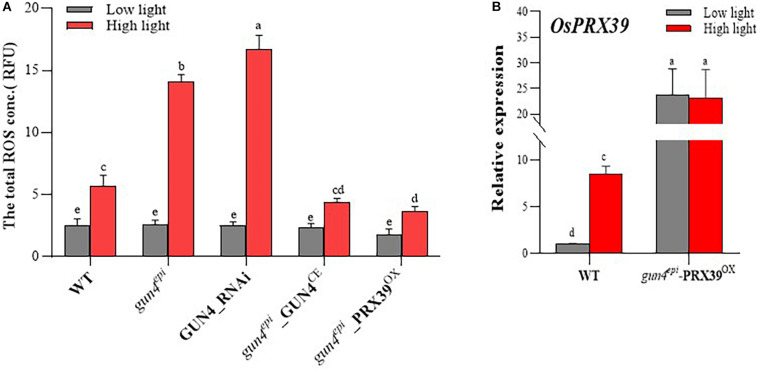
The ROS **(A)** and *OsPRX39* expression **(B)** levels in rice protoplasts. WT, wild-type variety LTB; *gun4*^*epi*^: HYB, an *OsGUN4* epigenetic mutant of LTB; GUN4_RNAi: LTB protoplasts transformed with an RNAi vector of *OsGUN4*; HYB_GUN4^CE^: HYB protoplasts with an *OsGUN4* complementary vector, *PRX39*^*OX*^: HYB protoplasts transformed with *OsPRX39* overexpression vector. Protoplasts were prepared from 35-day-old seedlings grown under low light (100 μmol m^–2^ s^–1^) and transformed with interference or complementary vectors. Transformed protoplasts were initially kept under dark for 1 h and then grown under low (100 μmol m^–2^ s^–1^) or high light (1000 μmol m^–2^ s^–1^) for 6 h, before subject to total RNA extraction. The expression level *of OsPRX39* are reported relative to that of the WT protoplasts grown at low light, which was assigned a value of 1. Values are means ± SD (*n* = 6) and analyzed for significant differences by two-way ANOVA followed by the Tukey’s multiple comparison test, *P* < 0.05.

To examine whether *OsGUN4* complementation and silencing affect ROS accumulation, the total ROS level in various types of protoplast was measured. Under LL conditions, no significant differences were observed among protoplasts with different *OsGUN4* genotypes, however, significant differences were observed after transferred to HL conditions (Figure 8A and Supplementary Table 10). Consistent with the results in seedlings (Figure 4A), a significantly greater total ROS level was observed in HYB protoplasts than its WT parent (Figure 8A). Importantly, the total ROS level was increased in GUN4_RNAi LTB protoplasts (to a level even significantly higher than that of HYB), while the total ROS level was reduced to the level of the WT in GUN4 complemented HYB protoplasts (*gun4*^*epi*^_GUN4^CE^).

To further examine the effect of repression of PRX genes on ROS accumulation, we overexpressed *OsPRX39* in HYB protoplasts (*gun4^*epi*^-PRX39^*OX*^*). The *OsPRX39* expression level in *gun4*^*epi*^_PRX39^*OX*^ protoplasts was high under both LL and HL conditions because it was driven by the Ubi promoter, and it showed a 2.73-fold increase as compared to that of WT under HL (Figure 8B and Supplementary Table 10). The total ROS level in *gun4*^*epi*^_PRX39^*OX*^ protoplasts was significantly reduced compared to the HYB protoplasts, and was even significantly lower that the WT LTB (Figure 8B).

## Discussion

The present study revealed that *OsGUN4* expression is light- and oxidative-stress responsive (Figure 1) and its complete knockout conferred a lethal phenotype (Supplementary Figure 2), suggesting OsGUN4 is indispensable for seed germination and seedling growth in rice. Up to 10 times ROS that of WT was accumulated in completely knocked out mutants, which might explain its lethal effect. We identified 8 PRX genes that were significantly downregulated in the *OsGUN4* mutant HYB and demonstrated that their expression was responsive to HL and H_2_O_2_ treatment in WT rice. However, the transcriptional response of the 8 PRX genes to HL and H_2_O_2_ treatment were either abolished or severely impaired in the *OsGUN4* mutants. Furthermore, we showed that there is a cause-and-effect relationship between *OsGUN4* mutation and altered expression and response of the 8 *PRX* genes to a light fluence shift in a protoplast expression system. Put together, we demonstrated that transcription of *OsGUN4* is regulated by oxidative stress and its response plays a critical role in ROS scavenging rather than ROS production, and we argue that the transcriptional regulation of the 8 *PRX* genes is facilitated by OsGUN4 via an as yet unknown signaling pathway in rice.

### *OsGUN4* Transcription Is Responsive to Oxidative Stress

We have made three significant observations:

(i) The expression of *OsGUN4* is sensitive to light intensity and oxidative stress (Figures 1, 7A). In photosynthetic organisms, the major sources of ROS production are PSI and PSII reaction centers (Asada, 2006; Møller et al., 2007), which is consistent with our observation that significantly more ROS is accumulated in WT seedlings grown under HL than LL conditions (Figure 7A). As we previously reported, there is an anti-oxidative element (ARE) in the promoter of *OsGUN4* (Li et al., 2014a), hence the observation that its transcription is responsive to oxidative stress was expected. Here, we further argue that the response of *OsGUN4* transcription to light intensity and oxidative stress might be governed by the same mechanism or by overlapping mechanisms.

(ii) *OsGUN4* transcription was low at night and increased rapidly at daybreak, and showed oscillation during the daytime (Figure 1A). The low expression at night is understandable because, at night, no photosynthesis takes place hence the ROS level also is low, while photosynthesis is active during the light period and the ROS level consequently increases. It is well-known that photosynthesis is reduced at midday in many crop plants including wheat (Sawada, 1978), rice (Ishihara and Saito, 1987), cotton (Pettigrew et al., 1990), maize (Tadashi and Theodore, 1999), and citrus (Hu et al., 2007). In a previous study, we observed the midday photoinhibition under natural field condition in the WT variety LTB (but not in HYB), with significantly reduced values of Fv/Fm and ϕPSIIaround 12 00 h (Wu et al., 2007). The underlying mechanism leading to midday depression of photosynthesis has been investigated from various aspects and at different levels, such as its relation to light-induced chlorophyll fluorescence changes (Wang et al., 2021) and chloroplast positioning (Maai et al., 2020), however, it is not yet fully understood. We observed a decrease in *OsGUN4* expression around midday in both WT varieties Nipponbare and LTB grown under HL conditions (Figure 1A), surprisingly similar to the reported midday depression of photosynthesis in LTB (Wu et al., 2007) and other varieties (Ishihara and Saito, 1987). It would be interesting to study whether depressed photosynthesis leads to a reduction in ROS content and subsequently to a downregulation of *OsGUN4* transcription, or vice versa.

(iii) We observed a significant increase in total ROS content in *OsGUN4* mutants. It is very interesting to note that while expression of *OsGUN4* is responsive to ROS in WT LTB, its mutation caused elevated ROS accumulation in HYB. Elevated accumulation could result from either increased production or reduced scavenging of ROS. Because the overall photosynthetic activity in LTB is known to be higher than in HTB than in HYB, particularly before the midday depression of photosynthesis (Wu et al., 2007), and there was indeed a lower ^1^O_2_ content, particularly under HL conditions (Figure 1C), therefore we argue that the it is very likely that the ROS scavenging capacity is impaired due to *OsGUN4* mutations.

### ROS Accumulation in *OsGUN4* Mutations May Result From Repression of PRX Genes

Plant cells deploy a delicate system to prevent or limit ROS production (Holt et al., 2004) and exquisite antioxidant system to scavenge excessive ROS in chloroplast (Gupta et al., 2015) via non-enzymatic (Havaux et al., 2007) and enzymatic reactions (Dietz, 2016).

In the present study, we identified 8 peroxidase encoding genes that were downregulated in HYB as compared with LTB, and further demonstrated that their transcriptional response to oxidative stress was abolished or repressed in *OsGUN4* mutant seedlings (Figure 6). Of particular importance is that the transcriptional repression of the 8 PRX genes was also observed in the mutant F_2_ seedlings, because it demonstrates that the differences between HYB and LTB is very likely due to the *OsGUN4* mutation. We further established the cause-and-effect relationship between *OsGUN4* mutation and its repression effect on transcription by silencing and complementing *OsGUN4* in protoplasts and determination of expression of the 8 PRX genes (Figure 7).

Peroxidases are important ROS scavengers in plants. It is therefore predictable that *OsGUN4* mutations such as downregulation (*gun4*^*epi*^) and knockout (*gun4-1*) would significantly impair the ROS scavenging system and result in ROS accumulation. Consistent with this prediction, the content of total ROS and H_2_O_2_ was significantly higher in the mutants than the WT, particularly under HL conditions (Figures 4A,B, 8).

The *gun4*^*epi*^ allele dramatically downregulates its transcription, but would still retain a very low level of OsGUN4 protein, while the *gun4-1* allele is expected to completely abolish its translation, which means that it would produce no OsGUN4 protein. The expected different mutational effects on *OsGUN4* expression was indeed further manifested at both the biochemical and phenotypical level. The *gun4-1/gun4-1* seeds could not germinate normally and conferred early death (Supplementary Figure 2), which is very likely due to the very high ROS accumulation t (Figure 4A). Moreover, significantly more ROS and H_2_O_2_ were accumulated in *gun4^*epi*^/gun4-1* seedlings than in *gun4^*epi*^/gun4^*epi*^* ones (Figures 4A,B), further supports the more severe effect of *gun4-1*. The different effects on ROS accumulation were well manifested on seedling growth: *gun4^*epi*^/gun4^*epi*^* grew much better than *gun4^*epi*^/gun4-1* seedlings (Figure 2).

### The Role of OsGUN4 in Retrograde Signaling and Regulation of PRX Genes, a Hypothesis

*Genomes Uncoupled 4* was identified as a regulator of chlorophyll synthesis and intracellular signaling in Arabidopsis (Larkin et al., 2003; Larkin, 2016). However, most of yet limited number of studies on GUN4 and its orthologs have been focusing on its regulatory function in tetrapyrrole biosynthesis, with only a few studies examined its role and mechanism in retrograde signaling.

In *Chlamydomonas reinhardtii*, Brzezowski et al. (2014) demonstrated that light-or dark-grown *gun4* mutant accumulates high levels of PPIX, and fails to downregulate mRNA levels of the tetrapyrrole biosynthesis and the photosynthesis-associated nuclear genes (PhANGs). While they proposed that GUN4 functions in ‘shielding’ PPIX, and most likely Mg-PPIX, by reducing reactivity with O_2_, and further suggested that “GUN4 seems to be involved in sensing elevated levels of these photoreactive tetrapyrrole intermediates, and contributing to ^1^O_2_-mediated retrograde signaling.” However, Tabrizi et al. (2016) argued for the opposite mechanism in that GUN4-PPIX is a ^1^O_2_ generator and proposed that the light-dependent ^1^O_2_ generation from GUN4-PPIX is the first step in retrograde signaling from the chloroplast to the nucleus. While the two studies suggested quite different roles of GUN4 in ^1^O_2_ generation, they suggested the same route of retrograde signaling, that is, EXECUTER 1 and/or EXECUTER 2 sense ^1^O_2_ level and regulate transcription of PhANGs.

In rice, we previously observed that downregulation of *OsGUN4* results in deregulated transcription of PhANGs and altered tetrapyrrole biosynthesis, as well as decreased ^1^O_2_ production and down-regulated ^1^O_2_-dependent retrograde signaling (Li et al., 2017). In the present study, we observed the same concentration changes of PPIX, Mg-PPIX and pchlide (Figures 3A–C) and ^1^O_2_ (Figure 4D) in both of *gun4^*epi*^/gun4^*epi*^* and *gun4^*epi*^/gun4-1* seedlings as in HYB. These results are consistent with the findings that OsGUN4 plays a role by promoting ^1^O_2_ generation and its deficiency is expected to impair ^1^O_2_-dependent retrograde signaling.

**FIGURE 9:**
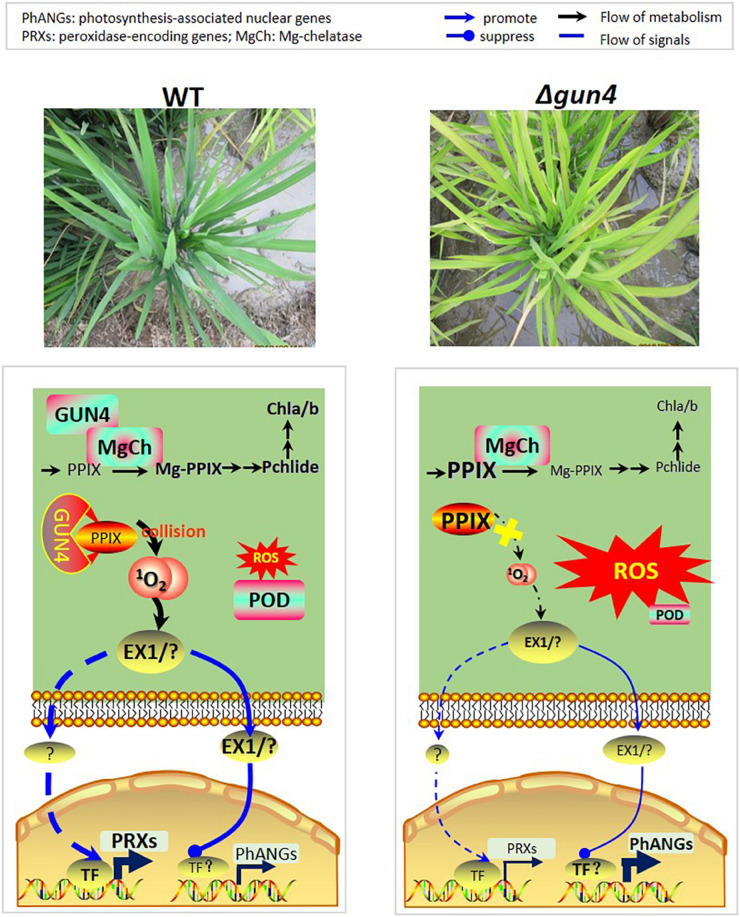
Model explaining the roles of OsGUN4 during oxidative stress. In WT plants, the ^1^O_2_ level increases with the progress of photosynthesis, and activate EXECUTER 1 and/or EXECUTER 2 sense ^1^O_2_ level and other transcription factor genes, such as BAP1, but greatly repress the transcription of PhANGs. The transcription factors further activate downstream target genes including PRX genes, and the peroxidases encoded by them together help scavenge ROS thus maintain a low ROS levels. However, in *gun4* mutants, the ^1^O_2_ level remains low due to lack of functional GUN4 hence the ^1^O_2_-mediated retrograde signaling pathway is not activated, and consequently, (1) a relatively high level of photosynthesis is maintained, which continuously generates ROS, (2) PRX genes are not activated hence ROS scavenging capacity remains low, which together result in the accumulation of excessive ROS.

Regarding the role of GUN4 in retrograde signaling, it has so far limited to the ^1^O_2_-dependent, EXECUTER 1 and/or EXECUTER 2 mediated transcription regulation of PhANGs. Our present study for the first time extended it to the regulation of PRX genes (Figure 9). While further studies are needed to establish the regulatory chain from GUN4 to PRX genes, we here propose that regulation of genes responsive for ROS scavenging, such as the PRX genes studied in the present study, is an essential function of the retrograde signaling pathway. We further envision the following working module: In WT plants, the ^1^O_2_ level increases with the progress of photosynthesis, and activate EXECUTER 1 and/or EXECUTER 2 sense ^1^O_2_ level and other transcription factor (TF) genes, such as BAP1. Some of the activated TFs repress the transcription of PhANGs after photosynthesis reaches to a high level, for example late morning, such that the photosynthesis is prohibited at midday. On the other side, some of the TFs activate genes including PRX genes, and the peroxidases encoded by them together help scavenge ROS thus maintain a low ROS level. However, in *gun4* mutants, the ^1^O_2_ level remains low due to lack of functional GUN4 hence the ^1^O_2_-mediated retrograde signaling pathway is not activated, and consequently, (1) a relatively high level of photosynthesis is maintained, which continuously generates ROS, (2) PRX genes are not activated hence ROS scavenging capacity remains low, which together result in the accumulation of excessive ROS (Figure 9). More studies are needed to validate our hypothesis and reveal the players leading to the activation of PRX genes downstream the ^1^O_2_-mediated signaling pathway.

## Conclusion

We here demonstrate that OsGUN4 is indispensable for rice seed germination and seedling growth, its deficiency results in inactivation of the ^1^O_2_ mediated signaling pathway. Consequently, the expression of PhANGs is deregulated and that of PRX genes is not activated, which together result in significant ROS accumulation in *OsGUN4* mutants.

## Data Availability Statement

The original contributions presented in the study are publicly available. This data can be found here: NCBI repository, https://www.ncbi.nlm.nih.gov/bioproject/PRJNA715818.

## Ethics Statement

The authors declare that the experiments were performed in compliance with the current laws of China.

## Author Contributions

Q-YS conceived the study and finished the final version. R-QL and MJ carried out the experimental analysis and data analysis. R-QL finished the first draft. IM and J-ZH revised the manuscript. All authors contributed to the article and approved the submitted version.

## Conflict of Interest

The authors declare that the research was conducted in the absence of any commercial or financial relationships that could be construed as a potential conflict of interest.

## Footnotes

1 ftp://ftp.ensemblgenomes.org/pub/plants/release-32/fasta/oryza_sativa/dna/Oryza_sativa.IRGSP-1.0.dna.toplevel.fa.gz
