# Supplementary material for: Mutations of the Genomes Uncoupled 4 Gene Cause ROS Accumulation and Repress Expression of Peroxidase Genes in Rice
Source: Front Plant Sci. 2021 Jun 11;12:682453. doi: 10.3389/fpls.2021.682453 (PMC8232891; doi:10.3389/fpls.2021.682453)
Supplement: Supplementary file 1 [file Data_Sheet_1.zip › Supplementary Figures.pdf]

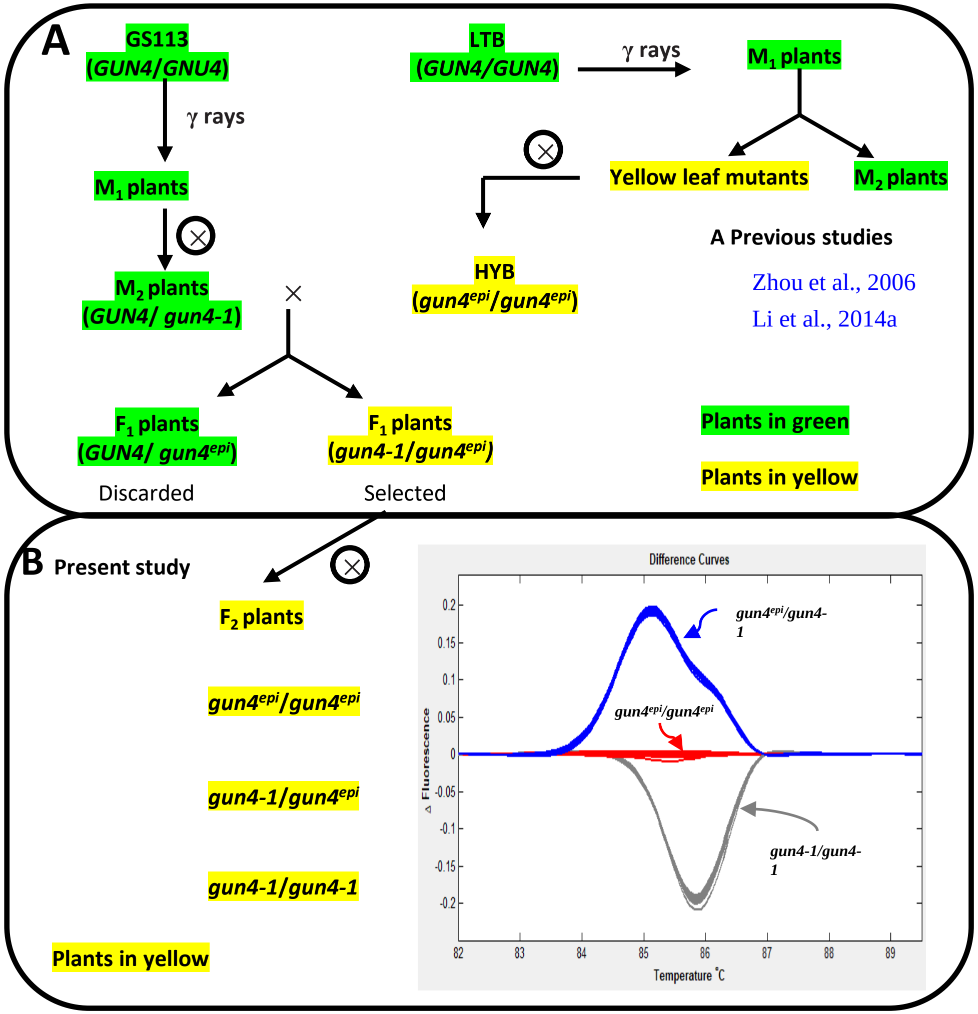

Supplementary Figure 1 | Background information on the breeding of *OsGUN4* mutants (A) and mutant plants used in the present study (B).

High resolution melting curve analysis was used for genotyping of F_2 plants derived from the *gun4-1/gun4*^{epi} F_1 plants (B, *right*).

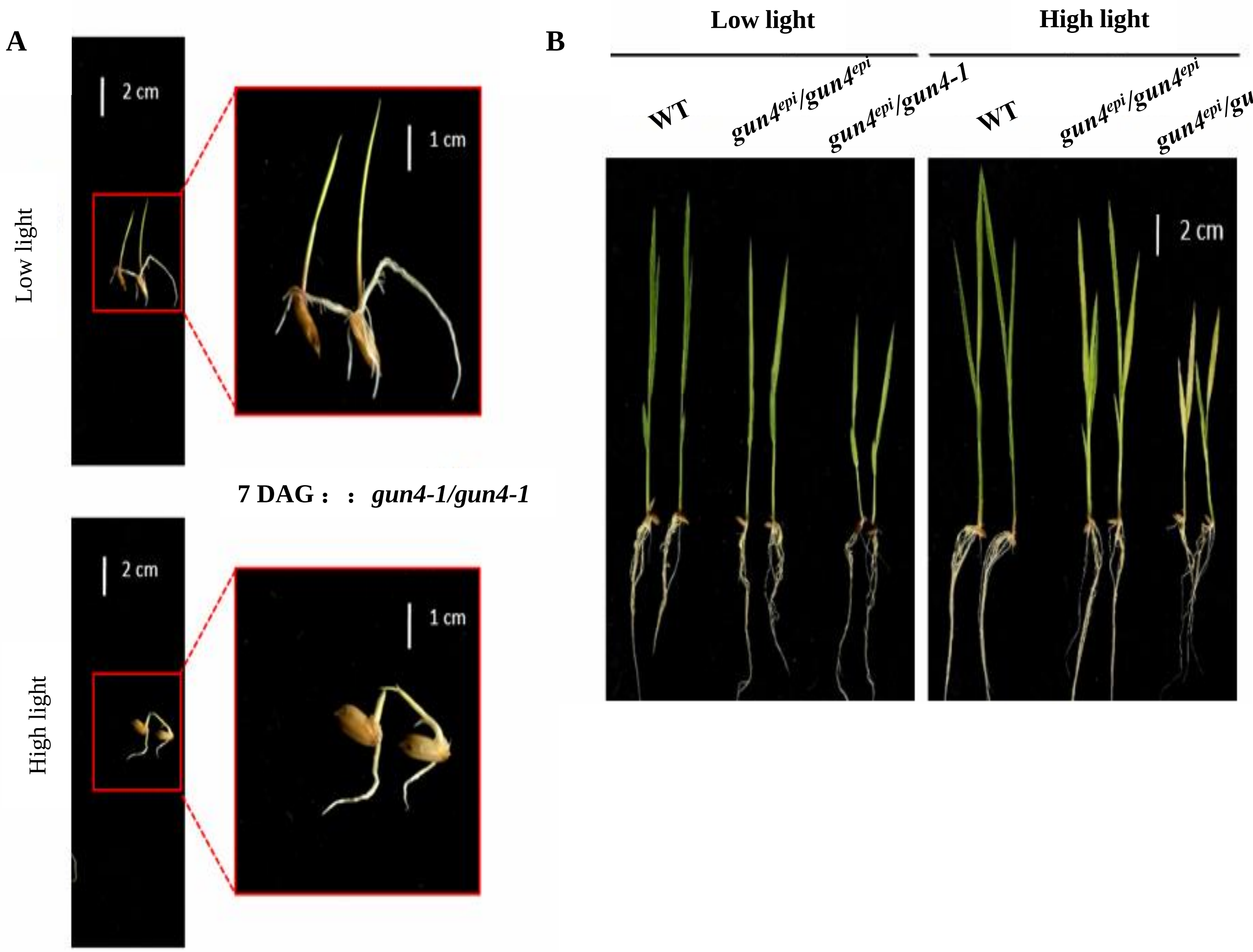

Supplementary Figure 2 | The phenotypes of the *OsGUN4* mutants under high light.

The phenotype and plant height of wild-type (WT; c.v. LTB) and three F_2 rice seedlings of the cross of HYB (*gun4^{epi}*/*gun4^{epi}*) and mutant GS113 (*gun4-1/gun4-1*) at (A) 7 and (B) 14 days after germination (DAG). Seedlings were grown under low (100 μmol m^{-2} s^{-1} photons) or high (1000 μmol m^{-2} s^{-1} photons) light conditions.
