# Supplementary material for: Mutations of the Genomes Uncoupled 4 Gene Cause ROS Accumulation and Repress Expression of Peroxidase Genes in Rice
Source: Front Plant Sci. 2021 Jun 11;12:682453. doi: 10.3389/fpls.2021.682453 (PMC8232891; doi:10.3389/fpls.2021.682453)
Supplement: Supplementary file 1 [file Data_Sheet_1.zip › Supplementary Tables 1-7.pdf]

Supplementary Table 1 | Primers used in this study

Name	Forward Primer（5’→3’）	Reverse Primer（5’→3’）
OsGUN4	TTACCGGCAGGCCGACGAGA	TGCCCAGGAGCTGTGTCCCT
OsUbiquitin	GCTCCGTGGCGGTATCAT	CGGCAGTTGACAGCCCTAG
OsPRX-L	GGCTCTTCAACTTCACCGGA	TGCAGAGATTAGCCATTACTCCA
OsPRX11	TACCGCGACCTCCTCAACTA	GAAGCCCGGCATGAACCT
OsPRX39	GCTGGCGAAGAGCTGCC	GTTCACCTCGATGTTGGCGA
OsPRX65	CTCCCGGACTCCACCTTCA	GGAGCAGTGGCCGACG
OsPRX81	CAGCATAGACATGGCCAAAGAG	AGTTCTTGCAGTCATGTGCCA
OsPRX86	ATCAGCATGTTCGGCAACCAG	GATGTTGCGCTCGGTGTAGAT
OsPRX89	TTCCACGACTGCTTCGTCAG	TCTCGACCACGCTCTTGATG
OsPRX125	AAGTTCGCCGCCCTCAAC	CTCCCCGTGTTGCTGAAGTT
OGUN4	AAGCTTTGGTCGTCTTCCCCTCCGATCTCC	GCCATGGGAAACAAGTGGAGATGATCAGCT
RGUN4	CGCGGCGCGACTTCACC	GGCAGGATGTGTGAAGATGTTGCCC
OPRX39	GCCATGGATGGGCGCTGTGGCTGCGGTT	GAATTC ACTTCCATTGACAAATCTG

Supplementary Table 2 l Two-way ANOVA test for the expression of *OsGUN4*

Difference	SS	df	MS	F	P-value	F crit
**7 DAG**						
Genotype	14.75370638	3	4.917902128	472.4158419	2.99062E-31	2.838745398
Growth condition	0.227476468	1	0.227476468	21.85148959	3.31931E-05	4.084745733
Interaction	0.68255759	3	0.227519197	21.85559412	1.52895E-08	2.838745398
Interior	0.416404506	40	0.010410113			
Total	16.08014495	35				
**21 DAG**						
Genotype	50.93538	2	25.46769	192.4124	7.74E-18	3.31583
Growth condition	8.467564	1	8.467564	63.97378	6.29E-09	4.170877
Interaction	16.9335	2	8.466752	63.96764	1.51E-11	3.31583
Interior	3.970798	30	0.13236			
Total	80.30724339	35				
**35 DAG**						
Genotype	866.1913	2	433.0957	176.8176	2.5E-17	3.31583
Growth condition	353.849	1	353.849	144.464	5.36E-13	4.170877
Interaction	707.3679	2	353.684	144.3966	4.02E-16	3.31583
Interior	73.48175	30	2.449392			
Total	2000.890005	35				

Supplementary Table 3 l Two-way ANOVA test for seedling height at 7, 21, 35 days after germination (DAG)

Difference	SS	df	MS	F	P-value	F crit
**7 DAG**						
Genotype	131.3886	3	43.79622	3259.253	1.69E-22	3.238872
Growth condition	2.567604	1	2.567604	191.0775	2.58E-10	4.493998
Interaction	2.067812	3	0.689271	51.29457	1.98E-08	3.238872
Interior	0.215	16	0.013438			
Total	136.2391	23				
**21 DAG**						
Genotype	316.3611	2	158.1806	2109.074	5.21E-16	3.885294
Growth condition	1.868889	1	1.868889	24.91852	0.000314	4.747225
Interaction	8.341111	2	4.170556	55.60741	8.53E-07	3.885294
Interior	0.9	12	0.075			
Total	327.4711	17				
**35 DAG**						
Genotype	341.3733	2	170.6867	802.1828	1.67E-13	3.885294
Growth condition	8.268889	1	8.268889	38.86162	4.36E-05	4.747225
Interaction	3.004444	2	1.502222	7.060052	0.009402	3.885294
Interior	2.553333	12	0.212778			
Total	355.2	17				

Supplementary Table 4 | Two-way ANOVA test for representative tetrapyrrole metabolites at 35 DAG

Difference	SS	df	MS	F	P-value	F crit
PPIX						
Genotype	0.21928	2	0.10964	48.84838	3.67E-10	3.31583
Growth condition	0.522714	1	0.522714	232.8876	1.11E-15	4.170877
Interaction	0.637446	2	0.318723	142.0024	5.04E-16	3.31583
Interior	0.067335	30	0.002244			
Total	1.446775099	35				
Mg-PPIX						
Genotype	0.030138	2	0.015069	56.49121	6.72E-11	3.31583
Growth condition	0.14248	1	0.14248	534.1392	1.14E-20	4.170877
Interaction	0.012229	2	0.006114	22.92177	9.08E-07	3.31583
Interior	0.008002	30	0.000267			
Total	0.192848168	35				
Pchlide						
Genotype	1586.053	2	793.0266	391.3099	3.22E-22	3.31583
Growth condition	368.5001	1	368.5001	181.8322	2.87E-14	4.170877
Interaction	1103.38	2	551.6902	272.2252	5.86E-20	3.31583
Interior	60.79784	30	2.026595			
Total	3118.731495	35				
Chlorophyll						
Genotype	5.294518	2	2.647259	144.0209	4.16E-16	3.31583
Growth condition	17.52449	1	17.52449	953.399	2.7E-24	4.170877
Interaction	0.180462	2	0.090231	4.908922	0.01431	3.31583
Interior	0.551432	30	0.018381			
Total	23.55090627	35				

Supplementary Table 5 l Two-way ANOVA test for the reactive oxygen species (ROS) level of seedlings at 7, 21, 35 DAG

Difference	SS	df	MS	F	P-value	F crit
**7 DAG**						
Genotype	5430.17	3	1810.057	3922.016	3.86E-23	3.238872
Growth condition	63.27068	1	63.27068	137.0944	2.93E-09	4.493998
Interaction	49.86636	3	16.62212	36.01667	2.41E-07	3.238872
Interior	7.384189	16	0.461512			
Total	5550.691	23				
**21 DAG**						
Genotype	453.1493	2	226.5746	4582.751	5E-18	3.885294
Growth condition	12.34069	1	12.34069	249.6056	2.14E-09	4.747225
Interaction	1.186085	2	0.593042	11.99501	0.001374	3.885294
Interior	0.593289	12	0.049441			
Total	467.2693	17				
**35 DAG**						
Genotype	628.0476	2	314.0238	1154.618	1.91E-14	3.885294
Growth condition	12.23601	1	12.23601	44.98996	2.17E-05	4.747225
Interaction	5.399771	2	2.699885	9.927068	0.002858	3.885294
Interior	3.263665	12	0.271972			
Total	648.9471	17				

Supplementary Table 6 | Two-way ANOVA test for concentration of H_2O_2 and $^1O_2$ level of seedlings at 35 DAG

Difference	SS	df	MS	F	P-value	F crit
H_2O_2						
Genotype	696.007	2	348.0035	421.7068	1.09E-22	3.31583
Growth condition	1311.272	1	1311.272	1588.985	1.52E-27	4.170877
Interaction	409.2441	2	204.622	247.9587	2.2E-19	3.31583
Interior	24.75679	30	0.825226			
Total	2441.280264	35				
$^1O_2$						
Genotype	47625500	2	23812750	160.3828	9.58E-17	3.31583
Growth condition	3722849	1	3722849	25.07401	2.28E-05	4.170877
Interaction	6507346	2	3253673	21.91403	1.36E-06	3.31583
Interior	4454234	30	148474.5			
Total	62309929.48	35				

Supplementary Table 7 l Summary of RNA-seq reads in wild-type LTB and its epigenetic *OsGUN4* mutant HYB

**Sample name**	**W1**	**W2**	**W3**	**M1**	**M2**	**M3**
Total reads	56110216	57165994	43157336	43869654	44697642	59938320
Clean reads	53892780	55802692	42314634	42099144	42994460	57897358
Clean bases (G)	8.08	8.37	6.35	6.31	6.45	8.68
Q20(%)	96.94	96.58	97.04	96.97	97.23	97.7
Total mapped	44062357 (81.76%)	45953764 (82.35%)	34940355 (82.57%)	34049684 (80.88%)	35110579 (81.66%)	49047507 (84.71%)
Multiple mapped	600371 (1.11%)	675652 (1.21%)	528478 (1.25%)	495590 (1.18%)	407376 (0.95%)	669156 (1.16%)
Uniquely mapped	43461986 (80.65%)	45278112 (81.14%)	34411877 (81.32%)	33554094 (79.7%)	34703203 (80.72%)	48378351 (83.56%)
Exon (%)	95.2	96.8	95.0	96.0	95.3	98.8
Intergenic (%)	3.5	2.3	3.5	2.6	2.5	0.6
Intron (%)	1.4	0.9	1.5	1.4	1.2	0.6
