# Supplementary material for: Mutations of the Genomes Uncoupled 4 Gene Cause ROS Accumulation and Repress Expression of Peroxidase Genes in Rice
Source: Front Plant Sci. 2021 Jun 11;12:682453. doi: 10.3389/fpls.2021.682453 (PMC8232891; doi:10.3389/fpls.2021.682453)
Supplement: Supplementary file 1 [file Data_Sheet_1.zip › Supplementary Tables 9-11.pdf]

Supplementary Table 9 l Two-way ANOVA test for gene expression under high light and exH_2O_2 treatment in 35 DAG seedlings

Difference	SS	df	MS	F	P-value	F crit
OsGUN4						
Genotype	1029.995802	2	514.9979009	8648.562967	1.41782E-27	3.554557146
Growth condition	61.4413463	2	30.72067315	515.9043865	1.28074E-16	3.554557146
Interaction	159.0901259	4	39.77253148	667.9158153	2.67638E-19	2.927744173
Interior	1.07185	18	0.059547222			
Total	1251.599124	26				
OsPRX-L						
Genotype	29.72687	2	14.86343	271.8138	3.57E-14	3.554557
Growth condition	1.104506	2	0.552253	10.09928	0.001146	3.554557
Interaction	3.292744	4	0.823186	15.05395	1.44E-05	2.927744
Interior	0.984283	18	0.054682			
Total	35.1084	26				
OsPRX11						
Genotype	14.59852	2	7.299259	147.5564	6.86E-12	3.554557
Growth condition	4.463513	2	2.231756	45.11553	9.73E-08	3.554557
Interaction	2.79922	4	0.699805	14.14674	2.17E-05	2.927744
Interior	0.890417	18	0.049468			
Total	22.75166852	26				
OsPRX39						
Genotype	36.80937	2	18.40468	212.6525	3E-13	3.554557
Growth condition	3.706807	2	1.853404	21.41471	1.74E-05	3.554557
Interaction	4.09027	4	1.022568	11.81501	6.95E-05	2.927744
Interior	1.557867	18	0.086548			
Total	46.16431296	26				
OsPRX65						
Genotype	10.42603889	2	5.213019444	400.6305415	1.19303E-15	3.554557146
Growth condition	34.84887222	2	17.42443611	1339.101331	2.63439E-20	3.554557146
Interaction	0.369088889	4	0.092272222	7.091297232	0.001303282	2.927744173
Interior	0.234216667	18	0.013012037			
Total	45.87821667	26				
OsPRX81						
Genotype	9.453238889	2	4.726619444	162.7115354	2.9853E-12	3.554557146
Growth condition	7.462005556	2	3.731002778	128.4379243	2.21423E-11	3.554557146
Interaction	4.605088889	4	1.151272222	39.63197654	1.08225E-08	2.927744173
Interior	0.522883333	18	0.029049074			
Total	22.04321667	26				
OsPRX86						
Genotype	8.742568519	2	4.371284259	70.94747678	2.90362E-09	3.554557146
Growth condition	50.0837463	2	25.04187315	406.4383848	1.05105E-15	3.554557146
Interaction	0.129037037	4	0.032259259	0.523579093	0.719699863	2.927744173

Interior	1.109033333	18	0.061612963			
Total	60.06438519	26				
OsPRX89						
Genotype	17.66745741	2	8.833728704	12.38767131	0.000413724	3.554557146
Growth condition	18.79566852	2	9.397834259	13.17872506	0.000298369	3.554557146
Interaction	3.425737037	4	0.856434259	1.20099071	0.344471995	2.927744173
Interior	12.83591667	18	0.713106481			
Total	52.72477963	26				
OsPRX125						
Genotype	13.88038519	2	6.940192593	112.1244596	6.90392E-11	3.554557146
Growth condition	11.13127963	2	5.565639815	89.9174408	4.27294E-10	3.554557146
Interaction	0.987925926	4	0.246981481	3.990186839	0.017263999	2.927744173
Interior	1.11415	18	0.061897222			
Total	27.11374074	26				

Supplementary Table 10 l Two-way ANOVA test for gene expression under high light treatment in protoplasts transformed with *OsGUN4* complementary and interference vectors

Difference	SS	df	MS	F	P-value	F crit
OsGUN4						
Genotype	393.3322	3	131.1107	40654.49	2.93E-31	3.238872
Growth condition	0.828817	1	0.828817	256.9974	2.81E-11	4.493998
Interaction	8.93075	3	2.976917	923.0749	3.95E-18	3.238872
Interior	0.0516	16	0.003225			
Total	403.1434	23				
OsPRX-L						
Genotype	10.28845	3	3.429482	487.6041	6.24E-16	3.238872
Growth condition	3.912338	1	3.912338	556.2565	7.43E-14	4.493998
Interaction	3.049079	3	1.01636	144.5061	8.46E-12	3.238872
Interior	0.112533	16	0.007033			
Total	17.3624	23				
OsPRX11						
Genotype	18.3051	3	6.1017	711.2229	3.13E-17	3.238872
Growth condition	2.561067	1	2.561067	298.5216	9.01E-12	4.493998
Interaction	2.6877	3	0.8959	104.4274	1.01E-10	3.238872
Interior	0.137267	16	0.008579			
Total	23.69113	23				
OsPRX39						
Genotype	17.79581	3	5.931937	365.9807	6.01E-15	3.238872
Growth condition	7.582504	1	7.582504	467.8152	2.85E-13	4.493998
Interaction	8.552113	3	2.850704	175.8789	1.85E-12	3.238872
Interior	0.259333	16	0.016208			
Total	34.18976	23				
OsPRX65						
Genotype	6.1409	3	2.046967	52.85336	1.59E-08	3.238872
Growth condition	23.32482	1	23.32482	602.2545	4E-14	4.493998
Interaction	1.605817	3	0.535272	13.82091	0.000105	3.238872
Interior	0.619667	16	0.038729			
Total	31.6912	23				
OsPRX81						
Genotype	3.832333	3	1.277444	44.57497	5.4E-08	3.238872
Growth condition	35.38082	1	35.38082	1234.573	1.42E-16	4.493998
Interaction	0.29165	3	0.097217	3.392265	0.043862	3.238872
Interior	0.458533	16	0.028658			
Total	39.96333	23				
OsPRX86						
Genotype	22.16475	3	7.38825	247.3054	1.3E-13	3.238872
Growth condition	42.40042	1	42.40042	1419.261	4.71E-17	4.493998
Interaction	0.892417	3	0.297472	9.957229	0.000607	3.238872
Interior	0.478	16	0.029875			

Total	65.93558	23				
OsPRX89						
Genotype	14.59435	3	4.864783	47.39964	3.49E-08	3.238872
Growth condition	35.47802	1	35.47802	345.6773	2.94E-12	4.493998
Interaction	2.195483	3	0.731828	7.130508	0.002952	3.238872
Interior	1.642133	16	0.102633			
Total	53.90998	23				
OsPRX125						
Genotype	24.51151	3	8.170504	463.794	9.27E-16	3.238872
Growth condition	28.18834	1	28.18834	1600.095	1.82E-17	4.493998
Interaction	3.196746	3	1.065582	60.48715	5.98E-09	3.238872
Interior	0.281867	16	0.017617			
Total	56.17846	23				

Supplementary Table 11 l Two-way ANOVA test for ROS accumulation under high light treatment in protoplasts

Difference	SS	df	MS	F	P-value	F crit
Genotype	463.696	4	115.924	340.0166	1.39E-35	2.557179
Growth condition	635.8968	1	635.8968	1865.148	2.99E-41	4.03431
Interaction	406.2488	4	101.5622	297.892	3.33E-34	2.557179
Interior	17.04682	50	0.340936			
Total	1522.888	59				
